# Supplementary material for: Research on an evolutionary game model and simulation of a cluster innovation network based on fairness preference
Source: PLoS One. 2020 Jan 13;15(1):e0226777. doi: 10.1371/journal.pone.0226777 (PMC6957165; doi:10.1371/journal.pone.0226777)
Supplement: Attachment — Submitted filename: Response to Reviewers.docx [file pone.0226777.s001.docx]

**Reviewer #1:** In this manuscript, the authors concern a cluster innovation network based on based on game theory and the fair preference theory, and investigate cooperation level and cooperation income in evolution process of cluster innovation network subjected to fair preference and return intensity. Although the numerical results are interesting, I do not recommend the manuscript for publication in its present form. I can reconsider my decision if a number of issues listed below are properly addressed.

**1.What is the BBV model? This manuscript investigates cluster innovation network constructed by BBV model, but there is no introduction about the model in the INTRODUCTION.**

**Answer:** The evolution model of weighted network was first established by Barrat, Barthelemy and Vespignani in 2004. It is usually called BBV model. It is a simple weight driven dynamic model, and generates statistical properties very similar to the real weight network, such as the law of network weight evolution with time, and the scale-free characteristics of strength distribution. Since the weighted scale-free network constructed by the weighted evolutionary BBV model was proposed, just like the BA scale-free network, it is gradually known. So in the introduction, the author did not introduce the BBV model in detail. In order to explain more clearly why BBV model is used to build cluster innovation network, the author makes the following modifications to this part of the text:

In addition, in the actual network evolution game process, the cooperation behavior of innovators is not only influenced by the network structure but also closely related to the intensity of the cooperative relationship between innovators[6], that is, the connection in the real network has the weight attribute. According to the existing research, a real network with connected weights has both the power-law distribution characteristics of the degree distribution and the power-law distribution characteristics of the strength distribution[7].

**2.The first sentence in 3.2 Construction of Game Model shows that whatever the strategy Sx chooses, the investment of enterprise x is always zero. Please check it.**

**Answer:** according to the reviewer’s opinion, the author found the writing error here, and has modified the paragraph as follows:

In the first round ($t_{n}=1$) of the public goods game, the degree value of enterprise $x$ is $k_{x}$, so if the enterprise $x$ chooses uncooperative strategy ($S_{\text{x}}=0$), the investment of enterprise $x$ in its neighborhood is 0; if enterprise $x$ chooses a cooperative strategy ($S_{\text{x}}=1$), it participates in $k_{x}+1$ neighborhoods centered on itself and neighbors, and its total investment is evenly distributed among $k_{x}+1$ neighborhoods.

**3.Figure.2(b) shows a nonmonotonic behavior of fc vs.** $\alpha$ **at r=2, while there is a monotonic behavior in Figure.3(b) for r=2. The results are inconsistent. Please check the numerical results.**

**Answer:** according to the reviewer’s opinion, author remade the simulation, redraws Figure 2 and figure 3, and corrects Figure 2 (b). The new simulation figure is as follows:

(a) w=0	(b) w=0.5	(c) w=1

Fig. 2 The effect of the adjustment coefficient on the level of enterprise cooperation in cluster innovation network under three mechanisms and different gain coefficients

(a) w=0	(b) w=0.5	(c) w=1

Fig. 3 The effect of the gain coefficient on the enterprise cooperation level in cluster innovation network under three mechanisms and different adjustment coefficients

**4.The manuscript is written very poorly, strongly lacking clarity in presentation. For example:**

(1)In Page2, “....and have find that in...” → “....and have found that in...”.

(2)In Page9, “...the greater the inhibitory effect on enterprise cooperation level are...” → “...the greater the inhibitory effect on enterprise cooperation level is...”.

(3)In Page10, “...improvement of preference in the terms of the breadth...” → “...improvement of preference in terms of the breadth...”.

(4)There have some inaccurate subscripts in the Eqs.(8) and (9).

(5) Some sentences are repeated, i.e., “... $S_{\text{x}}$ is the enterprise x game strategy ($S_{\text{x}}=1$ means cooperation, $S_{\text{x}}=0$ means non-cooperation)...”, which has been mentioned in the first paragraph in Construction of Game Model. Please check the full manuscript. A through improvement of English is necessary.

**Answer:** according to the reviewer’s opinion, the author revised (1)-(3) in the manuscript. For example: the previous income of the players^[30]^ and have found that in a static BA network….

the more significant the interactions between network structure and enterprise cooperative behaviors are, and the greater the inhibitory effect on enterprise cooperation level is.

This result shows that the improvement of preference in terms of the breadth and depth of cooperation…

Comments on Revision (4), The author referred to several references and rechecked formulas (8) and (9). No editorial errors were found in the subscripts of formulas (8) and (9). Each subscript is described here. In formula (8), The author referred to several references and rechecked formulas (8) and (9). No editorial errors were found in the subscripts of formulas (8) and (9). Each subscript is described here. In formula (8), the subscript $m_{x,\widehat{y}}$ represents the income of x in neighborhood $\widehat{y}$, the subscript $I_{x,\widehat{\mathrm{y}}}$ represents the input of x in neighborhood $\widehat{y}$, and the subscript $\widehat{y}=0$ in formula (9) represents the neighborhood centered on x (neighborhood $\widehat{y}$ refers to the neighborhood composed of neighbor enterprise $y$ and its directly connected enterprises). If you have any questions from the reviewer, please point out and I will revise it.

Comments on Revision (5), after careful comparison, the author has deleted similar sentences in the text and asked AJE to polish the full text, so as to improve the English level of the manuscript. Red font in English Revision

**Reviewer #2:** Review report for “Research on an Evolutionary Game Model and Simulation of a Cluster Innovation Network Based on Fairness Preference” by Li et al.

The cooperative environment of cluster innovation networks is an important issue that deserves further investigation. I found that this paper considers an interesting problem, the structure is well organized, and the paper is well written. However, there are some issues that hinder the acceptance of this paper in its current form. In the following, I would like to give some comments and suggestions, which may help the authors improve the quality of their paper.

**1, In the abstract, the authors directly go the introduction of methods and results. However, I think some introduction of general background regarding this topic should be added. Moreover, the authors are suggested to introduce their motivations after the background introduction. Furthermore, at the end of the abstract, it would be better to highlight the implication and application of their results.**

**Answer:** according to the reviewer’s opinion, the author added the background and purpose of the writing topic in the abstract, and added the significance of the paper research in the end. The added content is as follows:

At the beginning of the abstract: The cluster innovation network is an important part of regional economic development. In addition, the fairness preference of internal innovators in the processes of investment and benefit distribution are particularly important for curbing "free riding" and other speculative behaviors and for creating a good cooperation environment. Therefore, taking……

At the end of the abstract: Finally, we hope to improve the overall cooperation level and cooperation income of the network by deeply understanding the fair preferences of innovators in the processes of investment and benefit distribution, which is helpful for promoting the evolution and development of cluster innovation networks.

**2, In the introduction section, the authors present that “cluster innovation networks have the characteristics of a weighted scale-free network[8], so a simulation network based on the weighted evolution BBV model can simulate the real cluster innovation network well[9]”. I think it is not clear enough to the readers that whether cluster innovation networks have the characteristics of a weighted scale-free network. It would be better if the authors can explain more regarding this point.**

**Answer:** according to the reviewer’s opinion, in order to explain more clearly why BBV model is used to build cluster innovation network, the author makes the following modifications to this part of the text:

In addition, in the actual network evolution game process, the cooperation behavior of innovators is not only influenced by the network structure but also closely related to the intensity of the cooperative relationship between innovators[6], that is, the connection in the real network has the weight attribute. According to the existing research, a real network with connected weights has both the power-law distribution characteristics of the degree distribution and the power-law distribution characteristics of the strength distribution[7].

**3, On page 2, the authors present that “Some scholars also find that fixed static network structures such as rule networks[16], world networks[17] and scale-free networks[18] can also promote cooperation under certain conditions[19]”. I think “world networks[17]” should be “small-world networks[17]”. Moreover, in the follow sentence, “Santos[20] also found that the fairness preference of innovative subject cooperative behavior can greatly affect the level of cooperation in BA network”. It should be written as “Santos et al. [20] also found that xxx”**

**Answer:** according to the reviewer’s opinion, The author found that he had made some mistakes in the translation process, and now he has made some modifications to these two problems. Amend to read: Some scholars also find that fixed static network structures such as rule networks^[16]^, small-world networks^[17]^ and scale-free networks^[18]^ can also promote cooperation under certain conditions^[19]^. Santos et al^[20]^ also found that the fairness preference of innovative subject cooperative behavior can greatly affect the level of cooperation in BA network.

**4, In Figure, the sentences “****Previous Income、Cooperation Breadth” and “Current Investment、Cooperation Depth” should be revised. In English, there is no “、” but “,”. The authors should replace “、” by “,” in these two sentences.**

**Answer:** according to the reviewer’s opinion, the author changed "、" in Fig1 to "," and modified some words in Figure 1. Amend to read:

 Fig. 1 Evolution Analysis Framework of Cluster Innovation Networks under Fairness Preference

**5, In section 3.2 Construction of Game Model, the authors present that “it participates in** $k_{x}+1$ **neighborhoods centered on itself and neighbors, and its total investment is 0”. I think the total investment is 1 instead of 0. The authors should check this.**

**Answer**: after checking, the author found that there are some problems in this sentence, and has modified the paragraph as follows:

In the first round ($t_{n}=1$) of the public goods game, the degree value of enterprise $x$ is $k_{x}$, so if the enterprise $x$ chooses uncooperative strategy ($S_{\text{x}}=0$), the investment of enterprise $x$ in its neighborhood is 0; if enterprise $x$ chooses a cooperative strategy ($S_{\text{x}}=1$), it participates in $k_{x}+1$ neighborhoods centered on itself and neighbors, and its total investment is evenly distributed among $k_{x}+1$ neighborhoods.

**6, Before Equation (6), the sentence “as the center and directly connected enterprises) according to formula (6)”. I think Equation is more usually used than formula, and formula (6) should be replaced by “Equation (6)”. The same applies to the following sections, such as “Formula (7) shows that” and “Formula (8) shows that”. The authors are suggested to go through the paper and fix this problem.**

**Answer:** according to the reviewer’s opinion, the author has modified the “formula” in the full text to Eqs. (Equation) Since formula 6-11 has a large span in the text, it is not listed here. The modified part has been marked with red font.

**7, Below Equation (7), the sentence “Among these variables,** $\overline{D}_{x\text{，}\widehat{y}}(t_{n})\text{ }$ **is the normalized value of** $D_{x,\widehat{y}}(t_{n})$**,,** $t_{n}$ **is the number of rounds of the public goods game (only if all nodes in the network have a round of game to end the round of game)”. The readers may wonder which kind of normalization that the authors used. I thinks it would be better to add more details.**

**Answer:** according to the reviewer’s opinion, The author considers that the total investment of each enterprise is 1. At the same time, in order to ensure the linear change of investment, the author uses min max normalization method to standardize. The content of this paper is modified as follows:

Among these variables, considering that the total investment of each enterprise is 1, $D_{x,\widehat{y}}(t_{n})$ is normalized according to the min-max normalization method. $\overline{D}_{x\text{，}\widehat{y}}(t_{n})\text{ }$ is the normalized value of $D_{x,\widehat{y}}(t_{n})$, $t_{n}$ is the number of rounds of the public goods game (only if all nodes in the network have a round of game to end the round of game).

**8, For the following sentences, “the revenue of enterprise x(i)” and “denotes the degree value of enterprise y(i)”, it would be better to separate them into two sentences. It would be hard to distinguish x(i) from x and i.**

**Answer:** according to the reviewer’s opinion, according to the expert opinion, the author divides and adjusts the content of the sentence as follows:

…. and $m_{i,\widehat{y}}(t_{n-1})$ is the revenue of enterprise $i$ from neighborhood $\widehat{y}$ after the first round $t_{n-1}$ of the public goods game. and $m_{x,\widehat{y}}(t_{n-1})$ is the revenue of enterprise $x$ from neighborhood $\widehat{y}$ after the first round $t_{n-1}$ of the public goods game.

**9, In section 4.1 Simulation steps, the authors set an initial cooperation level as of 50%, i.e., the network cooperation density being 0.5. The authors may wonder how this parameter affects the results, and what happens if the initial cooperation level is set as 40% or 60%. Moreover, the cluster innovation network is with N=100 nodes, where I wonder how the network size affects the results. Maybe the authors want to add one or more figures to show the network size effects.**

**Answer:** according to the reviewer’s opinion, considering the below two reasons, the author decided not to increase the network cooperation density and network scale in this paper.

（1）before the experts put forward this opinion, the author has studied the influence of initial cooperation density and network scale on cooperation behavior, but found that the research conclusions are not innovative in this paper, so they are deleted and not reflected in the text. **A.** the emergence of cooperative behavior in the cluster innovation network is closely related to the game environment around the innovation subject. When adjusting the network cooperation density (0.4 or 0.6), it does not have a substantial impact on the change of cooperative behavior, only prolongs the network evolution time. **B**. before that, I have studied the network size of 50, 100, 200 and 500 under various influencing factors. It is found that the difference of the influence of network scale on cooperation behavior is only reflected in the number of iterations (evolution time). C. due to the figures 2.3 and 2.4 doesn’t reflect the trend of cooperation level over time. Therefore, the influence of network cooperation density and network scale on cooperation behavior can’t be reflected in this paper, so these two factors are put here, which makes the innovation slightly inadequate. Considering the actual simulation time and the dynamic evolution process of the network, the author finally selected the network scale of 100 for simulation research. However, according to the review experts, the research on the relationship between network scale and cooperation behavior over time has been reflected in another article "the game simulation research on knowledge transfer evolution of cluster innovation network under different network scales".

(2) this paper focuses on the impact of fair preferences of investment and payoff allocation on the cooperative behavior in the evolution process of cluster innovation network. In order to avoid too scattered research, the network cooperation density and network scale are not included in this study.

**10, In Figure 2 and Figure 3, I found a lot of fluctuations in the results. The authors present that “each data point is the average of simulation results after 50 independent experiments”. I think it would be better to increase the times of independent experiments. Maybe 500 is a better choice to avoid fluctuations and make the results more reliable. Moreover, the resolution of these two figures should be remarkably enhanced, and more detailed captions should be added to explain these figures.**

**Answer:** according to the reviewer’s opinion, and due to the limited simulation time, the author increased the number of independent experiments to 200 times, and took the average value of simulation results after 200 times. Therefore, the author re simulated, and the simulation results (Figure 2 and figure 3) of the average value are as follows:

(a) w=0	(b) w=0.5	(c) w=1

Fig. 2 The effect of adjustment coefficient on the level of enterprise cooperation in cluster innovation network under three mechanisms and different gain coefficients

(a) w=0	(b) w=0.5	(c) w=1

Fig. 3 The effect of gain coefficient on enterprise cooperation level in cluster innovation network under three mechanisms and different adjustment coefficients

**11, For the sentence “this paper uses Ruguo Fan [34] and Li [39] as references for the study of the weight coefficient, and implements three mechanisms to correspond to the three weight coefficients”. I think it would be better to revise “Ruguo Fan [34] and Li [39]” because it is not a typical reference style.**

**Answer:** according to the reviewer’s opinion, the author has deleted this part as required.

**12, In the reference section, many papers published in Chinese are referred, such as Refs [1],[7],[8],[17], [23],[33],and [35], however, these papers are usually invisible to the international community. I wonder if these works are follow ups of some very related international studies, and some of them can be replaced by references published in English journals. I will let the authors to decide.**

**Answer:** The author deleted the literature published in Chinese [1], [23], [33]. Chinese literature [5], [7], [8], [14], [17] and [35] are replaced by similar references published in some English journals, in order to meet the requirements of English journals.
