# Supplementary material for: Research on an evolutionary game model and simulation of a cluster innovation network based on fairness preference
Source: PLoS One. 2020 Jan 13;15(1):e0226777. doi: 10.1371/journal.pone.0226777 (PMC6957165; doi:10.1371/journal.pone.0226777)
Supplement: Attachment — Submitted filename: Response to Reviewers.doc [file pone.0226777.s002.doc]

**Reviewer #1:** The revised manuscript reads much better, is significantly improved and looks suitable for publication in Plos One.

**Reviewer #2**: Review report for “Research on an Evolutionary Game Model and Simulation of a Cluster Innovation Network Based on Fairness Preference”.
I would thank the authors for considering my previous comments and suggestions in revising their manuscript. I think the new version has been well improved. Before the consideration for publication, however, the authors are suggested to address the following two issues.

**1. For all figures,** **the labels of axis (as well as titles) are currently so small that it is very hard for the readers to pick up the information. The authors are suggested to remarkably increase the font size in all figures.**

**Answer:** according to the reviewer’s opinion, the author enlarges the label of axis (as well as the title) font of all Figure in the manuscript to ensure that readers can easily obtain information. Due to there are many Figure in manuscript, so I do not be displayed here. See the revised manuscript for details.

**2. Regarding my previous comment (9) “In section 4.1 Simulation steps, the authors set an initial cooperation level as of 50%, i.e., the network cooperation density being 0.5. The authors may wonder how this parameter affects the results, and what happens if the initial cooperation level is set as 40% or 60%. Moreover, the cluster innovation network is with N=100 nodes, where I wonder how the network size affects the results. Maybe the authors want to add one or more figures to show the network size effects”, I don’t think the authors tried their best to answer my question.**

**Firstly, in their reply, they presented that “When adjusting the network cooperation density (0.4 or 0.6), it does not have a substantial impact on the change of cooperative behavior, only prolongs the network evolution time”. However, I failed to find any figure in their reply that can support their claim.**

**Answer:** according to the reviewer’s opinion, the author put research on the influence of different network cooperation density on the evolution of cluster innovation network cooperation here to support the author's explanation. To ensure the scientific nature of the research, the author sets the weight coefficient (hybrid preference mechanism), the adjustment coefficient ($\alpha\text{=}1$，$\alpha\text{=2}$ and $\alpha\text{=3}$), three kinds of gain coefficient ($\text{r}=2$ $\text{r}=3$ and $\text{r}=4$), and three kinds of network cooperation density ($\text{p=}0.4$ $\text{p=}0.5$ and $\text{p=}0.6$) to carry out simulation analysis, to illustrate the influence of the network cooperation density on the evolution of cluster innovation network under different adjustment coefficients and gain capabilities. The simulation times are set to 100. After the system fully evolves to the stable stage, the average value of the last 30 cooperation densities is taken as $\text{P}$.

Figure 1 show the effect of the network cooperation density on the enterprise cooperation level in cluster innovation network under different adjustment coefficients. It can be seen from Figure 1 that under the same adjustment coefficient, with the increase of network cooperation density, the time from network evolution to stability is gradually longer, and the network evolution speed is gradually reduced. In addition, in the three adjustment coefficients, the simulation results are similar. Figure 2 show the effects of the network cooperation density on the enterprise cooperation level in cluster innovation network under different gain coefficients. the simulation results in Figure 2 are similar to those in Figure 1. Therefore, we have the same result.

Because of the similar simulation results under different parameters, the author thinks that it is not enough to study the influence of network cooperation density on the evolution of cluster innovation network cooperation. Therefore, the author thinks over and over again, and does not put the research results in the manuscript, and hopes that the reviewers will understand.

(a)$\alpha\text{=}1$ (b)$\alpha\text{=2}$ (c)$\alpha\text{=3}$

Fig. 1 The effect of the network cooperation density on the enterprise cooperation level in cluster innovation network under different adjustment coefficients

(a)$\text{r}=2$ (b)$\text{r}=3$ (c)$\text{r}=4$

Fig. 2 The effects of the network cooperation density on the enterprise cooperation level in cluster innovation network under different gain coefficients

**Secondly, the authors presented that “due to the figures 2.3 and 2.4 doesn’t reflect the trend of cooperation level over time. Therefore, the influence of network cooperation density and network scale on cooperation behavior can’t be reflected in this paper”. However, I could not find “figures 2.3 and 2.4”, and I am curious about the effects of the network scale and cooperation density other than the trend of cooperation level over time.**

**Answer:** Due to my writing mistakes, let you have misunderstanding figure 2.3 or 2.4, I apologize to you. What I want to explain is that in Figure 2, Figure 3, … and in Figure 7, the change trend of cooperation level over time is not reflected, and the influence of network cooperation density and network scale on cooperation behavior is not reflected in the manuscript. Therefore, it will be awkward to put these two factors here alone, and it will appear that innovation is a little inadequate.

**Thirdly, the authors presented that “the relationship between network scale and cooperation behavior over time has been reflected in another article "the game simulation research on knowledge transfer evolution of cluster innovation network under different network scales".”. However, I failed to find this referred article in web of science or using Google Scholar. I wonder if there is really a paper, and if this paper should be cited in the main text.**

**Answer:** first of all, here, the author apologizes to the reviewer. In the last reply to your opinion, I did not inform the reviewer of the relevant information of that manuscript. Here, Here, the author explains the manuscript to you.

The manuscript “Evolutionary Game Simulation of Knowledge Transfer in Industry-University-Research Cooperative Innovation Network under Different Network Scales” is currently under reviewed in 《scientific reports》. Due to it has not been uploaded, so it cannot be found in web of science or using Google Scholar. The manuscript was submitted at the end of August, and the overhaul comments were received on October 4st. On November 21st, the author submit the revised manuscript to 《scientific reports》. Among them, according to the reviewer’s opinion, in order to highlight the difference between enterprises and research institutions in knowledge transfer, the manuscript title has been slightly adjusted. The title “Evolutionary Game Simulation of Knowledge Transfer in Cluster Innovation Network under Different Network Scales” is modified to “Evolutionary Game Simulation of Knowledge Transfer in Industry-University-Research Cooperative Innovation Network under Different Network Scales”. Figure 3 is the proof of manuscript review in 《scientific reports》 after overhaul. Since the manuscript was revised before and after, the research on the relationship between the network scale and cooperative behavior has not changed much. Therefore, the conclusions of this study can be used for reference by reviewers. Considering that the manuscript is still in the process of review, the author will only present the relevant research contents about network scale and cooperation behavior in the manuscript “Evolutionary Game Simulation of Knowledge Transfer in Industry-University-Research Cooperative Innovation Network under Different Network Scales”, please understand. In the following text, red font is the conclusion of the study. There is a discussion about the influence of network structure on the evolution speed of knowledge transfer in cooperative behavior in the text.

Fig.3 Proof of manuscript review in scientific reports

**Evolutionary Game Simulation of Knowledge Transfer in Industry-University-Research Cooperative Innovation Network under Different Network Scales**

**Abstract**: This paper takes the industry-university-research cooperation innovation network constructed by the weighted evolutionary BBV model as the research object, which is based on bipartite graph and evolutionary game theory, and constructing the game model of knowledge transfer in the industry-university-research cooperation innovation network, by using the simulation analysis method and analyzing the evolution law of knowledge transfer in the industry-university-research cooperation innovation network under different network scales, three scenarios, the knowledge transfer coefficient and the knowledge reorganization coefficient. The results show that the increase of network size reduces the speed of knowledge transfer in the network. and the greater the average cooperation intensity of the nodes, the higher the evolution depth of knowledge transfer. Compared with university-research institutes, the evolution depth of knowledge transfer in enterprises is higher, and with the increase of network scale, the gap between the evolution depth of knowledge transfer between them is gradually increasing. Only when reward, punishment and synergistic innovation benefits are higher than the cost of knowledge transfer that can promote the benign evolution of industry-university-research cooperation innovation networks. Only when the knowledge transfer coefficient and the knowledge reorganization coefficient exceed a certain threshold will knowledge transfer behavior emerge in the network. With the increase of the knowledge transfer coefficient and the knowledge reorganization coefficient, the knowledge transfer evolutionary depth of the average cooperation intensity of all kinds of nodes is gradually deepening.

**Key words**: BBV model; Industry-University-Research Cooperative Innovation network; knowledge transfer; game model; simulation

**4.3 Simulation Results Analysis**

(1) The influence of the three scenarios on the knowledge transfer evolution of the industry-university-research cooperation innovation network under different network scales.

It can be found from Fig. 4 that the evolution simulation of knowledge transfer in the industry-university-research cooperation innovation network has similar evolution results under different network scales. In the same situation, along with the increase of network scale, the time of knowledge transfer evolving to a stable stage in the industry-university-research cooperation innovation network is gradually longer, and the evolution speed of knowledge transfer is gradually slower. This may be because the node degree, average weighted degree and shortest path in small-scale networks are relatively small, and the efficiency of information transmission is high. In large-scale networks, the more cooperative relationships there are among the nodes, the more uneven distribution and heterogeneity of the nodes, the more complex the process of revenue comparison and strategy learning among the nodes in the game process, the lower the efficiency of information transmission and the slower the speed of knowledge transfer^44^.

(a)$n=100$ (b)$n=200$ (c)$n=500$

Figure 4 Evolution simulation results of knowledge transfer in the industry-university-research cooperation innovation network under different network scales and three scenarios

(2) The influence of the knowledge transfer coefficient on the knowledge transfer evolution of the industry-university-research cooperation innovation network under different network scales.

On the basis of the situation in which the evolutionary depth of knowledge transfer in the industry-university-research cooperation innovation network is 0, the knowledge transfer coefficients $\alpha$ of advantage university-research institutes, advantage enterprise, general university-research institutes, and general enterprise obey the uniform distribution of $(0.05\text{+}i,0.15\text{+}i)$, $(0.10\text{+}i,0.20\text{+}i)$, $(0.15\text{+}i,0.25\text{+}i)$ and $(0.20\text{+}i,0.30\text{+}i)$ in turn. The values of $i$ are 0.0, 0.2, 0.4 and 0.6, respectively, to adjust the knowledge transfer coefficient $\alpha$ of all kinds of innovators, expressed by P1, P2, P3 and P4, respectively. The simulation results of the knowledge transfer evolution in the industry-university-research cooperation innovation network under different network scales and knowledge transfer coefficients are obtained, as shown in Fig. 5. Among them, Fig. 5 (a, b and c) respectively shows the simulation results of knowledge transfer evolution under three network scales, P1, P2, P3 and P4 represent the simulation evolution curves under four knowledge transfer coefficients.

From Fig. 5, it can be found that when the final results of network evolution converge to 0 under the same network scale, with the increase of knowledge transfer coefficient, the time for the evolution of the industry-university-research cooperation innovation network to be stable gradually becomes longer. This is because the increase of the knowledge transfer coefficient enhances the knowledge transfer ability, the knowledge transfer willingness and the knowledge absorption ability of the innovators, and the innovators’ knowledge transfer behavior preference gradually strengthens, so that the innovators’ cooperative behavior preference changes from a "non transfer" dominant direction to a "transfer" dominant direction. Under the same knowledge transfer coefficient, along with the increase of the network scale, the time for the evolution of the industry-university-research cooperation innovation network to stability gradually becomes longer. This is due to the increase of the network scale, the average path between innovators increases gradually, and the efficiency of information transmission decreases gradually. In the process of network games, innovators are faced with more complex revenue comparison and strategy selection, which leads to the slow evolution of knowledge transfer and the time from the evolution of the industry-university-research cooperation innovation network to stabilization gradually becomes longer. In addition, it can be found that only when $i$ is 0.6, that is, when the knowledge transfer coefficient is the maximum, the evolution result of knowledge transfer in the industry-university-research cooperation innovation network converges to 1. It shows that only when the knowledge transfer coefficient is higher than a certain threshold will the knowledge transfer behavior of innovators emerge in the network^48^.

(a)$n=100$ (b)$n=200$ (c)$n=500$

Figure 5 Evolution simulation results of knowledge transfer in the industry-university-research cooperation innovation network under different network scales and knowledge transfer coefficients

(3) The influence of the knowledge reorganization coefficient on the evolution of knowledge transfer in the industry-university-research cooperation innovation network under different network scales.

On the basis of the situation in which the evolutionary depth of knowledge transfer in the industry-university-research cooperation innovation network is 0, the knowledge reorganization coefficients $\beta$ of advantage university-research institutes, advantage enterprise, general university-research institutes, general enterprise obey the uniform distribution of $(0.02\text{+}j,0.03\text{+}j)$, $(0.015\text{+}j,0.025\text{+}j)$, $(0.01\text{+}j,0.02\text{+}j)$ and $(0.005\text{+}j,0.015\text{+}j)$ in turn. The values of $j$ are 0.01, 0.02, 0.03, and 0.04, respectively, to adjust the knowledge reorganization coefficients $\beta$ of all kinds of innovators, expressed by P1, P2, P3 and P4, respectively. The simulation results of the knowledge transfer evolution in the industry-university-research cooperation innovation network under different network scales and knowledge reorganization coefficients are obtained, as shown in Fig. 6. Among them, Fig. 6 (a, b and c) respectively shows the evolution results of knowledge transfer under three network scales. P1, P2, P3 and P4 represent the simulation evolution curves under four knowledge reorganization coefficients.

From Fig. 6, it can be found that when the final results of network evolution converge to 0 under the same network scale, with the increase of the knowledge reorganization coefficient, the time of knowledge transfer evolving to stability is gradually longer. However, when the final results of network evolution converge to 1, with the increase of the knowledge reorganization coefficient, the time of knowledge transfer evolving to stability is gradually shortened. This may be due to the increase of the knowledge reorganization coefficient, which makes the ability of innovators to understand, comprehend and apply knowledge gradually enhanced. The new knowledge acquired through digestion, absorption and reinnovation increases gradually, so that the knowledge transfer behavior preference of the innovator is gradually enhanced and then promotes the steady development of knowledge transfer in the industry-university-research cooperation innovation network. With the same knowledge reorganization coefficient, along with the increase of network scale, the time of knowledge transfer evolving to stability in the industry-university-research cooperation innovation network gradually becomes longer. This is because with the increase of the network scale, the level of knowledge application ability of innovators is not uniform, and the average path length between innovators is gradually increasing, which affects the transmission efficient knowledge information in the network, thus reducing the evolution speed of knowledge transfer and delaying the evolution time of knowledge transfer. In addition, it can be found from the graph that the final result of knowledge transfer evolution of the industry-university-research cooperation innovation network converges to 1 only when $j$ is 0.03. This also shows that only when the knowledge reorganization coefficient is higher than a certain threshold, the innovator has a certain ability of understanding, comprehending and applying the knowledge, so that the knowledge transfer behavior will emerge in the network.

(a)$n=100$ (b)$n=200$ (c)$n=500$

Figure 6 Evolution simulation results of knowledge transfer in the industry-university-research cooperation innovation network under different network scales and knowledge reorganization coefficients
